# Germline susceptibility from broad genomic profiling of pediatric brain cancers

**DOI:** 10.1093/noajnl/vdae099

**Published:** 2024-06-15

**Authors:** Elaine R Mardis, Samara L Potter, Kathleen M Schieffer, Elizabeth A Varga, Mariam T Mathew, Heather M Costello, Gregory Wheeler, Benjamin J Kelly, Katherine E Miller, Elizabeth A R Garfinkle, Richard K Wilson, Catherine E Cottrell

**Affiliations:** The Steve and Cindy Rasmussen Institute for Genomic Medicine, Nationwide Children’s Hospital, Columbus, Ohio, USA; Department of Pediatrics, The Ohio State University College of Medicine, Columbus, Ohio, USA; The Steve and Cindy Rasmussen Institute for Genomic Medicine, Nationwide Children’s Hospital, Columbus, Ohio, USA; Department of Pediatrics, The Ohio State University College of Medicine, Columbus, Ohio, USA; The Steve and Cindy Rasmussen Institute for Genomic Medicine, Nationwide Children’s Hospital, Columbus, Ohio, USA; Department of Pathology, The Ohio State University College of Medicine, Columbus, Ohio, USA; The Steve and Cindy Rasmussen Institute for Genomic Medicine, Nationwide Children’s Hospital, Columbus, Ohio, USA; The Steve and Cindy Rasmussen Institute for Genomic Medicine, Nationwide Children’s Hospital, Columbus, Ohio, USA; Department of Pathology, The Ohio State University College of Medicine, Columbus, Ohio, USA; The Steve and Cindy Rasmussen Institute for Genomic Medicine, Nationwide Children’s Hospital, Columbus, Ohio, USA; The Steve and Cindy Rasmussen Institute for Genomic Medicine, Nationwide Children’s Hospital, Columbus, Ohio, USA; The Steve and Cindy Rasmussen Institute for Genomic Medicine, Nationwide Children’s Hospital, Columbus, Ohio, USA; The Steve and Cindy Rasmussen Institute for Genomic Medicine, Nationwide Children’s Hospital, Columbus, Ohio, USA; Department of Pediatrics, The Ohio State University College of Medicine, Columbus, Ohio, USA; The Steve and Cindy Rasmussen Institute for Genomic Medicine, Nationwide Children’s Hospital, Columbus, Ohio, USA; The Steve and Cindy Rasmussen Institute for Genomic Medicine, Nationwide Children’s Hospital, Columbus, Ohio, USA; Department of Pediatrics, The Ohio State University College of Medicine, Columbus, Ohio, USA; The Steve and Cindy Rasmussen Institute for Genomic Medicine, Nationwide Children’s Hospital, Columbus, Ohio, USA; Department of Pathology, The Ohio State University College of Medicine, Columbus, Ohio, USA

**Keywords:** cancer predisposition, genetic susceptibility, hereditary cancer syndrome, molecular profiling

## Abstract

**Background:**

Identifying germline predisposition in CNS malignancies is of increasing clinical importance, as it contributes to diagnosis and prognosis, and determines aspects of treatment. The inclusion of germline testing has historically been limited due to challenges surrounding access to genetic counseling, complexity in acquiring a germline comparator specimen, concerns about the impact of findings, or cost considerations. These limitations were further defined by the breadth and scope of clinical testing to precisely identify complex variants as well as concerns regarding the clinical interpretation of variants including those of uncertain significance.

**Methods:**

In the course of conducting an IRB-approved protocol that performed genomic, transcriptomic and methylation-based characterization of pediatric CNS malignancies, we cataloged germline predisposition to cancer based on paired exome capture sequencing, coupled with computational analyses to identify variants in known cancer predisposition genes and interpret them relative to established clinical guidelines.

**Results:**

In certain cases, these findings refined diagnosis or prognosis or provided important information for treatment planning.

**Conclusions:**

We outline our aggregate findings on cancer predisposition within this cohort which identified 16% of individuals (27 of 168) harboring a variant predicting cancer susceptibility and contextualize the impact of these results in terms of treatment-related aspects of precision oncology.

**Key Points**

- Germline susceptibility contributes to ~16% of pediatric brain cancers.
- Comprehensive genomic profiling is required for identifying pathogenic variants.
- Germline cancer predisposition is increasingly critical for treatment decision-making.

**Importance of the Study**

In the next-generation sequencing era, our ability to expand the scope of inquiry in the setting of germline predisposition to cancer has yielded an improved appreciation of this contributor to cancer development, especially in pediatric CNS malignancies. Increasingly, this knowledge contributes to other aspects of care including precision of diagnosis and the clinical care of our youngest patients. As such, the translational importance of broad genomic testing that can accurately identify and inform clinical reporting of all types of variants contributing to cancer predisposition is obvious and impactful for patients and families. As reported recently, this impact now includes the identification of patients who have underlying mismatch repair deficiencies and an attendant high tumor mutational burden indicative of response to checkpoint blockade therapies.

Large-scale genomic characterization studies of pediatric CNS malignancies in the era of next-generation sequencing (NGS) have identified germline alterations that predispose to cancer onset. The breadth and scope of discovery of these foundational projects have identified both focal (single nucleotide and insertion–deletion) variants as well as copy number and structural alterations in around 400 cancer predisposition loci.^1^ Clinical sequencing of the germline in concert with tumor mutational profiling has historically been challenging for several reasons. This includes patient access for both the purpose of consent as well as acquisition of a germline specimen. Clinical and laboratory workflows may present challenges in genetic counseling, as well as access to phlebotomy or other germline specimen collection methods, which may be exacerbated if the laboratory primarily tests from archival tumor samples. The cost of assaying the germline sample, including challenges in reimbursement, can serve as an additional barrier. Furthermore, paired germline sequencing may be avoided due to the challenges of interpreting certain variants in the context of cancer risk, and hence the resulting difficulty both in communicating these results to families and patients and in translating variants of uncertain significance into the need for clinical surveillance regimens. Comparing the frequency of germline pathogenic variants across studies is complex, due to disparate cohort selection criteria and distributions of disease histology. For example, an analysis of 1022 medulloblastoma patients identified 6 medulloblastoma predisposition genes on the basis of rare variant burden analysis (*APC*, *BRCA2*, *PALB2*, *PTCH1*, *SUFU*, and *TP53*), and further noted that half of all patients with pathogenic germline variants were not recognized based on family history of cancer; however, the patients included in this study were largely sourced from tertiary referral centers with inherent referral bias.^2^ In juxtaposition, a population-based study of cancer predisposition in 280 pediatric glioma patients from California identified putatively pathogenic germline variants among 31 patients (11.1%) and had the benefit of eliminating referral bias, but only included individuals who self-reported as Latino or non-Latino white race/ethnicity in order to facilitate comparisons with public whole-exome sequencing control datasets.^3^ As germline results have become more important clinically and in determining treatment choices (*TP53* alterations in the setting of radiation therapy avoidance, for example), there has been a concomitant application of germline testing into the cancer precision oncology profiling rubric.

In 2018, we opened an institutional IRB-approved study (IRB17-00206) that pursued molecular profiling of DNA and RNA in the setting of pediatric cancer and hematologic malignancies. In this protocol, patients and families consented to undergo next-generation sequencing (NGS) of all protein-coding genes (the “exome”), in order to compare and identify variants in genomic DNA extracted from tumor and normal samples. RNA extracted from tumor was also sequenced and analyzed to identify the driver or diagnostic fusion genes and outlier gene expression. Due to the World Health Organization (WHO) adding methylation arrays to the clinical standard of diagnosis for CNS malignancies in 2021, we also adopted tumor DNA testing using Illumina methylation microarray technology and the DKFZ classifier^4^ for methylation-based classification of patients with CNS cancer. The overall workflow is outlined in Figure 1. Multi-platform testing provided precise diagnostic classification and permitted the identification of cancer-associated somatic single nucleotide and insertion–deletion (indel) variants, gene fusions, somatic and germline copy number alterations, and germline variants associated with cancer predisposition. In cases where a pathogenic or known pathogenic variant in any of these categories was identified by expert data evaluation, any variant deemed important for clinical decision-making or diagnosis (by the clinical care team) was subjected to clinical testing in our CAP-accredited and CLIA-certified laboratory, such that clinically confirmed variants could be reported into the patient’s medical record. Genetic counseling and cascade testing were provided to any patients and their families as a part of the IRB protocol when confirmatory clinical testing was obtained for a germline cancer predisposition variant.

**Figure 1.**
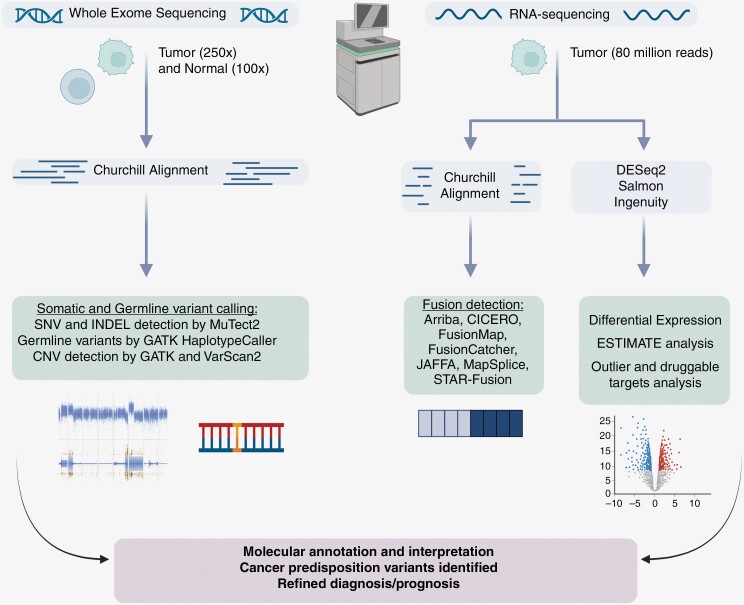
Workflow for Cancer/Germline Exome and Cancer RNA NGS Data Generation and Analysis. This figure illustrates the assays and analytics utilized in the NGS-based molecular characterization of pediatric CNS malignancies by our IRB-approved study.

## Materials and Methods

### Ethics Statement

All patients reported in this manuscript were enrolled on a study approved by the Nationwide Children’s Hospital IRB (IRB17-00206). Patients aged 18 or older were consented in person or by phone. Parental or legal guardian permission was obtained for patients under 18 years of age either in person or by phone. Assent was obtained from minors aged 9–17 years of age by written signature.

### Enhanced Exome Sequencing

DNA NGS libraries were prepared using input tumor or normal DNA and NEB Ultra II FS reagents (New England Biolabs). Target enrichment by hybrid capture was performed using the IDT xGen Exome Research Panel v1.0 enhanced or v2.0 with the xGenCNV Backbone and Cancer-Enriched Panels-Tech Access (Integrated DNA Technologies). Paired-end 151-bp reads were generated on the Illumina HiSeq 4000 or NovaSeq 6000 (Illumina Inc.). Data analysis was performed using Churchill.^5^ Reads were aligned to the human genome reference sequence (build GRCh37 or GRCh38) using BWA (v0.7.15) and refined according to community-accepted guidelines for best practices (https://gatk.broadinstitute.org/hc/en-us). Duplicate sequence reads were removed using samblaster-v.0.1.22, and local realignment was performed using the Genome Analysis Toolkit (v4.1.9).^6^ Somatic single nucleotide variation (SNV) and indel detection were performed using MuTect2.^7^ Germline variants were called using GATK’s HaplotypeCaller.^8^ Copy number variation (CNV) was assessed using a combination of GATK (v4.2.4.1) and VarScan2.^9^

### Variant Evaluation and Interpretation Processes

SNV, indel and copy number variants were identified and prioritized for further evaluation. In relation to cancer predisposition, disease-associated genes were curated from the published literature and genomic databases including those described by Zhang et al.,^1^ as well as genes with strong (Tier 1) or emerging (Tier 2) evidence of germline or somatic cancer association as documented in the Cancer Gene Census.^10^ Variants within the curated set of genes were assessed in relation to attributes such as population frequency, impact on the gene product, functional evidence, documentation in a reputable database (eg ClinVar), segregation, and other attributes as described in the standards and guidelines set forth by the American College of Medical Genetics and Genomics (ACMG) and the Association for Molecular Pathology for the interpretation of sequence variants.^11^ Germline variants were also examined in the tumor sample given that paired data were available to inform on the loss of heterozygosity, copy number alteration, or the presence of somatic variation associated with a second hit within the locus. CNV was evaluated in relation to the aforementioned curated gene lists, along with consideration of the mechanism of action of the involved locus in association with disease. Once published, the technical standard recommendations set forth by the ACMG and Cancer Genomics Consortium for the evaluation of cancer-associated CNV^12^ were further considered in our assessment. The study team discussed any positive research results indicative of a pathogenic or likely pathogenic variant in germline cancer predisposition genes with the oncologist of record, who then ordered clinical confirmation of the variant to be performed by our CAP/CLIA laboratory. Upon clinical confirmation, the patient and family were apprised of the test result in our Cancer Predisposition Clinic and counseled according to appropriate cascade testing.

Enrolled subjects had the option to consent to the return of secondary findings including medically actionable genetic variants, as well as carrier variants. Medically actionable secondary findings were reported in accordance with the policy statement set forth by the ACMG (v2.0 or 3.0) in the setting of clinical exome and genome sequencing.^13,14^ An internally curated list of genes with relevance to carrier status (for reproductive counseling) were also analyzed (*ASPA*, *BLM*, *CFTR*, *FANCC*, *G6PD*, *GBA*, *HBB*, *HEXA*, *IKBKAP*, *MCOLN1*, and *SMPD1*) to enable the return of pathogenic or likely pathogenic findings.

## Results and Discussion

### Cohort Findings in Cancer Predisposition Syndromes

**Figure 2.**
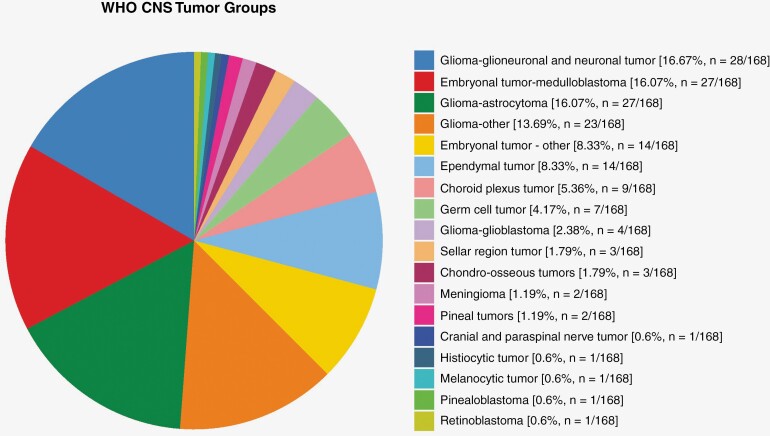
CNS diagnosis by WHO classification. This figure shows the proportions and numbers of different WHO classification-based diagnoses for the CNS malignancies characterized by molecular profiling analyses in this cohort.

Clinically significant germline alterations were identified in 23.7% (38/168) of patients with CNS malignancies enrolled on this protocol. This includes cancer predisposition in 16% (27/168), carrier alterations in 4.8% (8/168), and medically actionable secondary findings in 2.4% (4/168) of patients. One patient did not undergo germline sequencing due to sample unavailability. The majority of alterations detected were single nucleotide variants-36 SNVs were found in 18 different genes as noted in Table 1. In contrast, only 4 copy number variants were identified, 3 of which were detected in the *SMARCB1* gene (Table 2). Germline exome sequencing for our cohort of patients with pediatric CNS malignancies identified cancer predisposition in 16% of cases, which is within the 8–18% range reported by other studies of sporadic pediatric malignancies.^1,15–19^ Interestingly, by evaluating only the CNS patients in these referenced studies, the percentages of identified germline pathogenic/likely pathogenic alterations in cancer predisposition genes exhibited a wide range, between 4.5% and 31%. This discrepancy emphasizes the importance of methodologic transparency in cohort assembly and in the breadth of genomic inquiry, as biases in individual cohorts or in the amount of the genome being assayed can produce increased variability in estimating germline contributions to pediatric CNS malignancies. Proportions of WHO CNS tumor classification-based diagnoses in our cohort are shown in Figure 2.

**Table 1.** Germline Single Nucleotide Variants Identified by Enhanced Exome Sequencing

Gene	SNV	Cancer Predisposition	Carrier/ Medically Actionable	Comments
*CHEK2* (NM_007194.4)	c.470T > C:p.Ile157Thr c.1100del:p.Thr367MetfsTer15 c.444 + 1G > A:p.?	✔ ✔ ✔		
*CFTR* (NM_000492.4)	c.1521_1523del:p.Phe508del (*n* = 4) c.1624G > T:p.Gly542Ter		✔ ✔	
*GBA* (NM_000157.4)	c.1192C > T:p.Arg398*		✔	
*GLA* (NM_000169.3)	c.1087C > T p.Arg363Cys		✔	
*GPR161* (NM_001375883.1)	c.972T > A:p.Tyr344Ter	✔		
*G6PD* (NM_001360016.2)	c.844G > C:p.Asp282His		✔	
*HBB* (NM_000518.5)	c.20A > T:p.Glu7Val		✔	
*KCNQ1* (NM_000218.3)	c.1085A > G p.Lys362Arg		✔	
*NF1* (NM_001042492.3)	c.1318C > T:p.Arg440Ter (*n* = 2) c.4977_4980del: p.Lys1661GlyfsTer36 c.7189G > A:p.Gly2397Arg	✔ ✔ ✔		
*NF2* (NM_000268.4)	c.1198del:p.Gln400ArgfsTer26	✔		
*MSH6* (NM_000179.3)	c.3939_3957dup:p.Ala1320SerfsTer5 (*n* = 2)	✔		*n* = 1 (Homozygous); *n* = 1 (Heterozygous)
*MYO18B* (NM_032608.7)	c.6768del p.Leu2257SerfsTer16 c.6660_6670del p.Arg2220SerfsTer74		✔ ✔	Both alterations noted in a single patient
*PALB2* (NM_024675.4)	c.3170_3175delCTTCAGinsAATCA:p.Val10	✔		
*PMS2* (NM_000535.6)	c.137G > T:p.Ser46Ile c.1A > G:p.Met1?	✔ ✔		
*PTPN11* (NM_002934.5)	c.179G > C:p.Gly60Ala c.209A > G:p.Lys70Arg c.922A > G:p.Asn308Asp	✔ ✔ ✔		
*SDHA* (NM_004168.4)	c.1579del p.Arg527ValfsTer20	✔		
*SMARCB1* (NM_003073.5)	c.644del:p.Pro215LeufsTer14 c.771_772dup: p.(Ser258CysfsTer10)	✔ ✔		Mosaic
*TP53* (NM_000546.6)	c.743G > A:p.Arg248Gln c.916C > T:p.Arg306Ter c.548C > G:p.Ser183Ter c.722C > G:p.Ser241Cys	✔ ✔ ✔ ✔		

**Table 2.** Copy Number Germline Variants Identified by Enhanced Exome Sequencing

Gene	Copy Number Variant	Size and Disease Association
*HNF1B*	17q12 deletion	1.31 Mb; renal cysts and diabetes syndrome
*SMARCB1*	22q11.21-q11.23 loss	2.68 Mb deletion; ATRT, Distal 22q deletion syndrome
*SMARCB1*	22q11.22q11.23	1.34 Mb deletion; ATRT
*SMARCB1*	22q11.22q11.23	1.88 Mb deletion;ATRT

Genetic cancer predisposition was identified in 27 patients (Table 3 and Supplementary Table 1). These diagnoses included rhabdoid tumor predisposition syndrome (RTPS), Li Fraumeni syndrome (LFS), Lynch syndrome, neurofibromatosis types 1 and 2, NS, and tumor predisposition syndrome 4 (TPDS4). RTPS was detected in 5 patients with atypical teratoid/rhabdoid tumor (ATRT); notably, 4 of these individuals presented with synchronous renal rhabdoid tumors.^20,21^ LFS was detected in 2 patients with choroid plexus carcinoma, as well as in individuals with medulloblastoma and anaplastic pleomorphic xanthroastrocytoma. Neurofibromatosis type 1 (NF1) was detected in 4 patients, of which 2 were diagnosed with pilocytic astrocytoma. Noonan syndrome (NS) was identified in 3 patients, all of whom were diagnosed with glioneuronal tumors. *CHEK2* alterations associated with TPDS4 were noted in 3 patients with disparate diagnoses: medulloblastoma, subependymal giant cell astrocytoma, and atypical meningioma. These cases have been previously reported.^22^ Alterations in DNA repair genes associated with mismatch repair cancer syndrome and Lynch syndrome were identified in 4 cases. Of these, 2 patients diagnosed with glioblastoma were found to have heterozygous germline alterations in *PMS2* consistent with Lynch syndrome 4. Germline *MSH6* gene alterations were detected in 2 patients with high-grade gliomas, 1 with heterozygous *MSH6* (NM_000179.3) p.Ala1320SerfsTer5 consistent with Lynch syndrome 5, and the other with homozygous *MSH6* (NM_000179.3) p.Ala1320SerfsTer5, diagnostic for mismatch repair cancer syndrome 3 (also described as constitutional mismatch repair deficiency (CMMRD) syndrome). Germline alterations in *GPR161*, *NF2*, *PALB2*, and *SDHA* were identified in single patients.

**Table 3.** Cancer Predisposition and CNS Malignancy Diagnoses

Cancer Predisposition	Age at Cancer Diagnosis (y)	Sex	Germline Alteration	Malignancy
Familial Breast-Ovarian Cancer 5	0.5	M	NM_024675.4(PALB2):c.3170_3175delCTTCAGinsAATCA:p.Val1059fsTer18	Pilocytic astrocytoma
Li Fraumeni Syndrome	5	M	NM_000546.6(TP53):c.548C > G:p.Ser183Ter	Anaplastic pleomorphic xanthoastrocytoma
Li Fraumeni Syndrome	4	M	NM_000546.6(TP53):c.722C > G:p.Ser241Cys	Medulloblastoma, SHH subgroup
Li Fraumeni Syndrome	1.2	F	NM_000546.6(TP53):c.743G > A:p.Arg248Gln	Choroid plexus carcinoma
Li Fraumeni Syndrome	0.92	M	NM_000546.6(TP53):c.916C > T:p.Arg306Ter	Choroid plexus carcinoma
Lynch Syndrome 4	14	F	NM_000535.6(PMS2):c.1A > G:p.Met1?	Glioblastoma
Lynch Syndrome 4	13	M	NM_000535.6(PMS2):c.137G > T:p.Ser46Ile	Glioblastoma
Lynch Syndrome 5	13	F	NM_000179.3(MSH6):c.3939_3957dup:p.Ala1320SerfsTer5	Diffuse midline glioma H3 K27M mutant
Medulloblastoma Predisposition Syndrome	1.5	M	NM_001375883.1(GPR161):c.972T > A:p.Tyr344Ter	Medulloblastoma, SHH subgroup
Mismatch Repair Cancer Syndrome 3	8	F	NM_000179.3(MSH6):c.3939_3957dup:p.Ala1320SerfsTer5	Adult-type diffuse high-grade glioma, IDH wildtype
Neurofibromatosis type 1	11	F	NM_001042492.3(NF1):c.1318C > T:p.Arg440Ter	Anaplastic astrocytoma
Neurofibromatosis type 1	0.5	F	NM_001042492.3(NF1):c.1318C > T:p.Arg440Ter	Plexiform neurofibroma
Neurofibromatosis type 1	4	M	NM_001042492.3(NF1):c.4977_4980del:p.Lys1661GlyfsTer36	Pilocytic astrocytoma
Neurofibromatosis type 1	12	F	NM_001042492.3(NF1):c.7189G > A:p.Gly2397Arg	Pilocytic astrocytoma
Neurofibromatosis type 2	12	M	NM_000268.4(NF2):c.1198del:p.Gln400ArgfsTer26	Meningioma
Noonan Syndrome	9	F	NM_002834.5(PTPN11):c.209A > G:p.Lys70Arg	Glioneuronal neoplasm with worrisome molecular features, not elsewhere classified
Noonan Syndrome	21	M	NM_002834.5(PTPN11):c.179G > C:p.Gly60Ala	Rosette-forming glioneuronal tumor
Noonan Syndrome	16	M	NM_002834.5(PTPN11):c.922A > G:p.Asn308Asp	Rosette-forming glioneuronal tumor
Pheochromocytoma/Paraganglioma Syndrome 5	4	F	NM_004168.4(SDHA):c.1579del p.Arg527ValfsTer20 (carrier)	Yolk sac tumor
Rhabdoid Tumor Predisposition Syndrome	0.83	F	NM_003073.5(SMARCB1):c.644del:p.Pro215LeufsTer14	Atypical teratoid/rhabdoid tumor and rhabdoid tumor of the kidney
Rhabdoid Tumor Predisposition Syndrome	13	M	NM_003073.5(SMARCB1):c.771_772dup: p.Ser258CysfsTer10 (mosaic ~20% variant allele frequency)	Atypical teratoid/rhabdoid tumor
Rhabdoid Tumor Predisposition Syndrome/Distal 22q deletion syndrome	5	F	22q11.21-q11.23 loss (including *BCR, IGL, MAPK1, SMARCB1*)	Atypical teratoid/rhabdoid tumor
Rhabdoid Tumor Predisposition Syndrome	0.17	F	1.34 Mb deletion on 22q11.22-q11.23 (including *SMARCB1*)	Atypical teratoid/rhabdoid tumor and rhabdoid tumor of the kidney^a^
Rhabdoid Tumor Predisposition Syndrome	0.13	F	1.88 Mb deletion of 22q11.22q-11.23 (including *SMARCB1*)	Atypical teratoid/rhabdoid tumor and rhabdoid tumor of the kidney
Tumor Predisposition Syndrome 4	6	M	NM_007194.4(CHEK2):c.470T > C:p.Ile157Thr	Medulloblastoma, Group 4
Tumor Predisposition Syndrome 4	9	M	NM_007194.4(CHEK2):c.1100del:p.Thr367MetfsTer15	Atypical meningioma
Tumor Predisposition Syndrome 4	7	M	NM_007194.4(CHEK2):c.444 + 1G > A:p.?	Subependymal giant cell astrocytoma

^a^Tumor sequencing for this sample was performed on rhabdoid tumor only.

The incorporation of comprehensive genomic profiling using multiple modalities inclusive of both germline and disease-involved tissue, poised our study well to expand on existing knowledge in this field. Our cohort included syndromic germline variants for which cancer development constitutes a significant morbidity such as LFS (≥70% lifetime risk)^23^ and CMMRD, a syndrome with cancer often occurring within the first decade of life.^24^ This is in contrast to that of NS, a collectively common autosomal dominant genetic disorder (1:1000–1:2500),^25^ which demonstrates an estimated cancer risk of 4% by age 20.^26^ NS, in addition to the collective RASopathies, has displayed some genotype–phenotype correlations, with certain genes and variants more commonly associated with cancer, particularly in hematologic malignancy.^27^ Interestingly, 3 patients with CNS malignancies in our cohort also harbored NS-associated germline pathogenic variants. Two of 3 NS diagnoses were made from paired exome sequencing analysis carried out through this translational protocol, thereby allowing refined surveillance, management, and counseling.

### Carrier Alterations and Medically Actionable Secondary Findings

Carrier alterations were reported for 8 patients (8/168, 4.8%). Four patients were heterozygous for *CFTR* (NM_000492.4) p.Phe508del, and 1 patient presented with a *CFTR* (NM_000492.4) p.Gly542Ter alteration. Heterozygous alterations in *G6PD*, *HBB*, and *GBA* were each found in a single patient. These genes are associated with glucose-6-phosphate dehydrogenase (G6PD) deficiency, sickle cell disease, and Gaucher disease, respectively.

Medically actionable secondary findings were noted in 4 cases. *GLA* (NM_000169.3) p.Arg363Cys conferring Fabry disease was identified in a female patient with a low grade glioneuronal tumor and was clinically confirmed. A 17q12 deletion incorporating the *HNF1B* gene associated with renal cysts and diabetes syndrome was identified, was clinically confirmed, and the patient is followed by nephrology. One patient with ganglioglioma was discovered to have a *KCNQ1* (NM_000218.3) p.Lys362Arg alteration that is frequently documented as likely pathogenic (ClinVar Variation ID: 52953) in association with prolonged QT syndrome. Klippel–Feil syndrome was identified in 1 patient found to carry compound heterozygous variants in *MYO18B* (NM_032608.5, p.Arg2220SerfsTer74 and p.Leu2257SerfsTer16). Both variants were clinically confirmed, and cascade testing led to a similar diagnosis for a sibling in utero.^28^

### Importance of Germline Comparator Data

The inclusion of paired germline exome data demonstrates significant advantages, particularly in the interpretation of tumor-associated variation and disease diagnosis refinement. For example, 1 patient we studied was previously diagnosed with an IDH wildtype adult-type diffuse high-grade glioma by an outside laboratory that performed panel-based tumor somatic profiling of 101 genes. Their clinical results reported a high mutational burden, and listed 15 gene variants, 11 of which were at near-to slightly reduced heterozygous allele frequency, challenging their interpretation as either somatic or germline. This patient carried a clinical diagnosis of Neurofibromatosis 1 due to the observation of numerous cafe au lait spots, inguinal and axillary freckling, and Lisch nodules. However, prior reference laboratory clinical testing of *NF1* and *SPRED1* through NGS and deletion/duplication testing was negative from blood. Our paired exome analysis identified a homozygous germline *MSH6* variant (NM_000179.2(MSH6):c.3939_3957dup:p.Ala1320SerfsTer5) in tandem with a region of homozygosity of 2p, resulting in a diagnosis of CMMRD. This patient was also noted to have 12% of the genome comprised of long-contiguous stretches of homozygosity consistent with a genomic finding of consanguinity. The clinical overlap of CMMRD and NF1 phenotypes has been previously described.^29^ Notably, the *MSH6* germline variant we identified was reported as a somatic variant with a VAF of 37% in the tumor-only panel-based testing at the outside laboratory. This VAF was likely underestimated as the *MSH6* variant consists of a 19bp duplication which results in a high percentage of soft clipping in aligned reads and a reduction in overall aligned reads in comparison to SNVs. The numerous other variants with near-to slightly reduced heterozygous VAF were further clarified as being germline or somatic by our paired exome testing, allowing us to accurately identify the etiology of germline susceptibility, and to assign and interpret somatic variation. Achieving an accurate diagnosis enabled appropriate genetic counseling and cancer surveillance to occur for this patient and family. Diagnostic criteria to aid in the identification and definition of CMMRD have been proposed^30,31^ and increasing evidence suggests that immune checkpoint inhibitors may be impactful in treatment and improved survival amid cancers with high mutational burden in this population.^32^ While immune checkpoint inhibitors were not implemented for CNS malignancy in this patient, treatment with pembrolizumab was later initiated after a subsequent diagnosis of T-lymphoblastic lymphoma. While this patient, unfortunately, died secondary to infectious complications, the identification and application of targeted treatment were enabled by germline genomic testing. This case illustrates the impact that cancer, as an inherited disease, can have and emphasizes the clear advantage of exome-wide, paired germline studies.

### Potentiating Discovery of New Gene-Disease Associations

The paired exome approach also enables the identification of novel cancer predisposition genes and variants or those not previously associated with a given cancer type. The American College of Medical Genetics has established best practices for the report of germline variation for individuals who undergo cancer-related testing.^33^ Care in clinical interpretation needs to be taken to understand if such variation is a secondary finding, or is potentially contributory to the cancer under study. One such example from our cohort involved a patient diagnosed with a yolk sac tumor who harbored a germline variant in *SDHA* (NM_004168.4) predicted to encode a premature stop of translation (p.Arg527ValfsTer20). Germline pathogenic variation among succinate dehydrogenase complex genes (*SDHA, SDHB, SDHC*, and *SDHB*) is associated with Hereditary Paraganglioma–Pheochromocytoma Syndrome, which may result in the presentation of a variety of tumors (paraganglioma, pheochromocytoma, gastrointestinal stromal tumor, and pulmonary chondroma).^34^ The observed *SDHA* variant was considered pathogenic in ClinVar by multiple clinical laboratories (Variant ID:653810) and demonstrated an enriched variant allele frequency in the tumor (60%) relative to the germline (43%), lending evidence for its potential disease association with the yolk sac tumor. A recent study suggests that the cancer association for *SDHA* pathogenic germline variation may be broader than previously known, including neuroblastoma, melanoma, renal cell carcinoma, breast cancer, endometrial cancer, colon cancer, and prostate cancer.^35^ Furthermore, there are rare reports of succinate dehydrogenase gene variation described in germ cell tumors including in mediastinal germ cell tumor and in testicular seminoma, the latter of which demonstrated loss of heterozygosity of a nonsense variant in *SDHD*.^36,37^ More recently, co-occurrence of a germline pathogenic *SDHA* variant with a somatic *KIT* mutation was reported in a CNS germinoma, with no detected loss of heterozygosity or second hit in *SDHA*.^38^ The authors speculated that the *SDHA* variant might be disease-modifying and contribute to tumor aggressiveness but recognized that further evidence is necessary to establish a relationship between succinate dehydrogenase complex gene variants and germ cell tumors.

In such examples, it is critical to correlate germline and somatic variation along with histopathologic findings, additional molecular studies, and clinical presentation, to best interpret genetic contribution to disease. When disease–gene relationships remain uncertain, the detection of a secondary cancer predisposition variant in an individual with a seemingly unrelated cancer lends value for counseling, the opportunity for cascade testing, and at times may result in altered management or surveillance. As genomic profiling studies increasingly utilize testing of a germline comparator, cumulative evidence will shed additional light on disease–gene relationships, emphasizing the importance of publishing case report findings and sharing data broadly.

### Exomes Permit Re-evaluation of Emergent Germline Susceptibility Genes

Another benefit of exome sequencing in this patient population is that emerging gene targets with disease association can be readily evaluated without expanding the gene panel. This was demonstrated by the discovery of a *GPR161* (NM_001375883.1) p.Tyr344Ter germline alteration in a patient with medulloblastoma. The association between *GPR161* germline alterations and medulloblastoma predisposition syndrome was not described until 2020,^39^ 2 years after our protocol had opened to enrollment. However, with refinement to our variant prioritization strategies, such emerging or expanding disease–gene relationships can be incorporated readily into our variant review workflow.

### Limitations of the Study

The main limitation of this translational study is that it was founded on the premise of clinician nomination for individuals with rare or treatment-refractory cancers, rather than on the basis of systematic selection. While this enabled our team to focus on cases of significant clinical interest, it also may have led to selection bias in our cohort. Despite this limitation, our findings support much of the commonly referenced literature surrounding germline cancer predisposition in this population, while highlighting opportunities for further research, improved definition of germline susceptibility loci, and collaboration.

Our findings underscore the importance of paired germline and tumor testing in the pediatric cancer patient population and the potential benefits of exome sequencing in quickly incorporating emerging genes of clinical importance.

## Supplementary Material

vdae099_suppl_Supplementary_Table_S1
