# Supplementary material for: Germline susceptibility from broad genomic profiling of pediatric brain cancers
Source: Neurooncol Adv. 2024 Jun 15;6(1):vdae099. doi: 10.1093/noajnl/vdae099 (PMC11259010; doi:10.1093/noajnl/vdae099)
Supplement: vdae099_suppl_Supplementary_Table_S1 [file vdae099_suppl_supplementary_table_s1.docx]

**Supplementary Table 1. Cancer Predisposition Syndromes**

| **Cancer Predisposition Syndrome** | **Gene(s) Associated** | **Description** |
| --- | --- | --- |
| Familial Breast-Ovarian Cancer 5 | *PALB2* | Individuals with heterozygous pathogenic alteration in *PALB2* carry increased risk of breast and ovarian cancer. |
| Li Fraumeni Syndrome | *TP53* | Autosomal dominant hereditary cancer syndrome characterized by early onset of tumors, multiple tumors in one individual, and multiple affected family members. Most common tumors include soft tissue sarcoma, osteosarcoma, breast cancer, brain tumors, leukemia, and adrenocortical carcinoma. |
| Lynch Syndrome 4  (Hereditary Nonpolyposis Colorectal Cancer Type 4) | *PMS2* | Primarily associated with development of early-onset colorectal cancer; also correlated with development of other epithelial tumors, including endometrial cancer, stomach cancer, and ovarian cancer. Conferred by heterozygous alteration in *PMS2* and associated with autosomal dominant inheritance. |
| Lynch Syndrome 5  (Hereditary Nonpolyposis Colorectal Cancer Type 5) | *MSH6* | Caused by a heterozygous alteration in *MSH6* and characterized by development of colorectal cancer and/or extracolonic cancer, including endometrial cancer, usually in mid-adulthood. |
| Medulloblastoma Predisposition Syndrome | *GPR161*  *SUFU*  *BRCA2*  *ELP1* | Associated with development of medulloblastoma in childhood. |
| Mismatch Repair Cancer Syndrome 3 | *MSH6* | Autosomal recessive childhood cancer predisposition syndrome characterized by brain tumors, hematologic malignancy, and gastrointestinal tumors. Dermatologic features include multiple café-au-lait spots, axillary freckling, and occasionally, Lisch nodules. |
| Neurofibromatosis type 1 | *NF1* | Autosomal dominant disorder conferring increased susceptibility to development of both benign and malignant tumors, including neurofibromas and malignant peripheral nerve sheath tumors. Associated with dermatologic findings including Lisch nodules, café-au-lait spots, and fibromatous tumors of the skin. |
| Neurofibromatosis type 2 | *NF2* | Characterized by development of non-malignant tumors, particularly meningiomas and acoustic schwannomas; unlike NF1, patients tend to have relatively few skin lesions. |
| Noonan Syndrome | *PTPN11* | Genetic disorder associated with short stature, dysmorphic facial features, congenital heart defects, skeletal malformations, and bleeding diathesis. Inheritance is autosomal dominant and confers predisposition to certain malignancies, including neuroblastoma, rhabdomyosarcoma, and leukemia. |
| Pheochromocytoma/Paraganglioma Syndrome 5 | *SDHA* | Autosomal dominant disorder characterized by development of paragangliomas or pheochromocytomas, normally in adulthood. |
| Rhabdoid Tumor Predisposition Syndrome | *SMARCB1*  *SMARCA4* | Predisposes to development of rhabdoid tumors in childhood; autosomal dominant inheritance. Also associated with susceptibility to other central nervous system tumors, including medulloblastoma and choroid plexus carcinomas. |
| Tumor Predisposition Syndrome 4 | *CHEK2* | Associated with an increased risk of breast cancer and likely moderate risk association with prostate cancer and colorectal cancer. |
